# Using artificial intelligence and statistics for managing peritoneal metastases from gastrointestinal cancers

**DOI:** 10.1093/bfgp/elae049

**Published:** 2024-12-30

**Authors:** Adam Wojtulewski, Aleksandra Sikora, Sean Dineen, Mustafa Raoof, Aleksandra Karolak

**Affiliations:** Department of Machine Learning, H. Lee Moffitt Cancer Center and Research Institute, 12902 Magnolia Drive, Tampa FL 33612, United States; Department of Computer and Information Science and Engineering, University of Florida, 432 Newell Dr, Gainesville, FL 32611, United States; Department of Medicine, Medical University of Warsaw, Żwirki i Wigury 61, 02-091 Warszawa, Poland; Department of Gastrointestinal Oncology, H. Lee Moffitt Cancer Center and Research Institute, 12902 Magnolia Drive, Tampa FL 33612, United States; Division of Surgical Oncology, Department of Surgery, City of Hope National Medical Center, 1500 East Duarte Road Duarte, CA 91010, United States; Department of Machine Learning, H. Lee Moffitt Cancer Center and Research Institute, 12902 Magnolia Drive, Tampa FL 33612, United States; Department of Gastrointestinal Oncology, H. Lee Moffitt Cancer Center and Research Institute, 12902 Magnolia Drive, Tampa FL 33612, United States

**Keywords:** artificial intelligence, machine learning, deep learning, statistics, peritoneal metastasis, gastrointestinal cancers

## Abstract

**Objective:**

The primary objective of this study is to investigate various applications of artificial intelligence (AI) and statistical methodologies for analyzing and managing peritoneal metastases (PM) caused by gastrointestinal cancers.

**Methods:**

Relevant keywords and search criteria were comprehensively researched on PubMed and Google Scholar to identify articles and reviews related to the topic. The AI approaches considered were conventional machine learning (ML) and deep learning (DL) models, and the relevant statistical approaches included biostatistics and logistic models.

**Results:**

The systematic literature review yielded nearly 30 articles meeting the predefined criteria. Analyses of these studies showed that AI methodologies consistently outperformed traditional statistical approaches. In the AI approaches, DL consistently produced the most precise results, while classical ML demonstrated varied performance but maintained high predictive accuracy. The sample size was the recurring factor that increased the accuracy of the predictions for models of the same type.

**Conclusions:**

AI and statistical approaches can detect PM developing among patients with gastrointestinal cancers. Therefore, if clinicians integrated these approaches into diagnostics and prognostics, they could better analyze and manage PM, enhancing clinical decision-making and patients’ outcomes. Collaboration across multiple institutions would also help in standardizing methods for data collection and allowing consistent results.

## Introduction

Among all gastrointestinal cancers, which altogether represent the deadliest cancers [1], colorectal and gastric comprise the second leading cause of cancer-related deaths in the United States and the fourth globally [2]. These cancers are alarming because of their high propensity to metastasize without evident symptoms, making them more difficult to treat, especially in advanced stages. Further, one out of three individuals with metastatic gastrointestinal cancer [3] experiences peritoneal metastases (PM), which further complicates treatment strategies. The limited efficacy of therapeutic approaches for PM has motivated researchers and clinicians, and as a result, promising therapies have emerged.

Treating PM is complicated. The disease is complex and heterogenous, and traditional diagnostics are fraught with subjectivity and variability. In addition, data integration during manual assessments is limited (i.e. a focus on a single type of data). Because of these complications, using complementary approaches such as artificial intelligence (AI) is critical. AI could help clinicians to identify more effective tools for detecting PM and diagnosing and treating patients. Machine learning (ML) and deep learning (DL) AI models, increasingly used across various medical fields, are essential for (i) timely analysis of large volumes of data; (ii) automation of routine tasks; and (iii) identification of complex patterns that may be missed through manual assessments. ML and DL models not only modernize current decision-making processes but also deliver results that are often as accurate as, or superior to, traditional methods [4–6]. Classical ML models are algorithms that learn patterns from data to make predictions or decisions without being explicitly programmed. DL models are a subset of ML that use neural networks with many layers (thus *deep*) to learn complex patterns in large datasets. Both ML and DL prediction models play important roles at all stages of cancer research, from as early as assessing the risk of cancer development, to predicting metastatic occurrence, and to determining patient outcomes and treatment options. Considering the complex nature of PM caused by gastrointestinal cancers, which limits the efficacy of conventional methods (e.g. in evaluating intratumor or interpatient heterogeneity, detecting small or diffuse metastatic lesions, or addressing resistance), novel methods and treatments identified by the applications of AI and advanced statistical models are worth considering.

### AI in PM: balancing precision and generalizability

As discussed in this review, AI unquestionably can improve PM treatments for gastrointestinal cancers. AI can assist oncologists with making accurate prognoses of PM for patients with gastrointestinal cancers, among other tasks. However, because of the complexity of PM, oncologists must choose the right approach—they must consider the trade-off between a model’s performance and its transparency. AI approaches can elucidate PM by delivering supporting information about feature significance and other valuable details; however, AI often lacks generalizability. By contrast, DL approaches are generalizable; they are driven by image data, which generates universal models to accommodate a variety of contexts. Despite valuable insights, genomic data analyses using AI for PM are still largely unexplored. While the technology evolves, the studies discussed here show the promise of AI for earlier detection, diagnosis, and prognosis of PM.

### Challenges in implementing AI for PM preclinical research and patient care

Despite the promise of AI, implementing it in clinical settings for PM presents several challenges. Because PM cannot be fully understood through a single approach, integrating multiple models is needed to address the complexity of the PM condition and its underlying mechanisms. Yet, among the identified studies, only a few articles that integrated multiple models were identified for PM (see Methods). These models (i) addressed highly heterogeneous PM-related questions; (ii) used various sets of data; and (iii) used a high variety of AI models. This dearth of studies with multiple models highlights the need for more collaborative research. Among the discussed articles, only a small group of these studies showed overlapping models—some shared the data type, but none shared the data beyond external set model validation. Thus, to better understand PM, it is vital to promote additional studies that use the same models discussed here. However, studies with multiple models would allow for even more meaningful comparisons and facilitation of models transfer across different research institutions, ultimately improving data-integration strategies and advancing the field of PM.

Because data privacy and security are paramount, AI systems handling large volumes of data that include protected health information are at risk of compromising patient confidentiality. Integrating AI technologies with electronic health records can also increase the risk of exposing patient information. Additionally, the approval processes and ethical concerns, e.g. algorithmic bias and fairness, are critical for successful AI acceptance. Limited computational resources may restrict access to these technologies, especially in resource-constrained environments where large-scale data storage (often requiring terabytes) exceeds the available capacity. A potential solution could be establishing centralized data repositories to facilitate data sharing. Additionally, proper training is essential to address resistance and skepticism from experimental researchers confident to adopt and effectively use these advanced tools at the preclinical level. Addressing these challenges is essential to fully realize the benefits of AI for improving PM outcomes and patient care.

**Figure 1:**
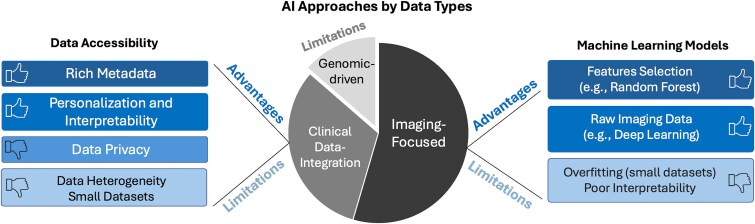
**Illustration of the advantages and limitations of existing AI approaches for peritoneal metastases (PM) arising from gastrointestinal cancers.**  Center: AI approaches for PM are categorized into three domains: Imaging-focused, Genomic-driven, and Clinical Data-Integrated. The genomic-driven section specifically addresses limitations in current datasets and models relevant to gastrointestinal cancer PM, as discussed in this review. Right: Imaging-focused approaches demonstrate strengths, such as effective feature selection (e.g. random forest models) and processing raw imaging data (e.g. deep learning models). However, limitations include poor model interpretability, and overfitting risks due to small datasets, which are particularly significant in PM imaging datasets. Left: Clinical data integration enables patient-specific predictions and enriches available data, aiding in personalized treatment strategies. Key challenges include data privacy concerns and small, heterogeneous clinical datasets that restrict model robustness and predictive accuracy in PM applications.

In summary, using the articles from the field of PM, we discuss how AI and statistics can benefit patients at increased risk for PM and patients already suffering from it. We present and discuss data through comprehensive tables to make it accessible for a variety of researchers, including computational, experimental, and clinical. Despite the limited and heterogeneous nature of the available publications on AI for PM, our structured approach allows for quick access to the (i) relevant AI method, (ii) data type and size, and (iii) clinical question investigated. We emphasize the unique and most recent contributions of each methodology to important clinical applications. AI analyses of PM are predominantly driven by imaging and clinical data, which provide detailed and immediate insights into the status of PM. In contrast, genomic studies remain understudied, highlighting a gap in integrating comprehensive genetic information into AI models for PM, especially in the early stages of PM (Fig. 1). Figure 1 underscores the necessity of developing large-scale, standardized datasets and innovative methods to overcome these limitations while leveraging the unique advantages of each AI-driven approach. We further explore these challenges and limitations in the sections, Discussion and Conclusions and Future Directions.

## Methods

**Figure 2:**
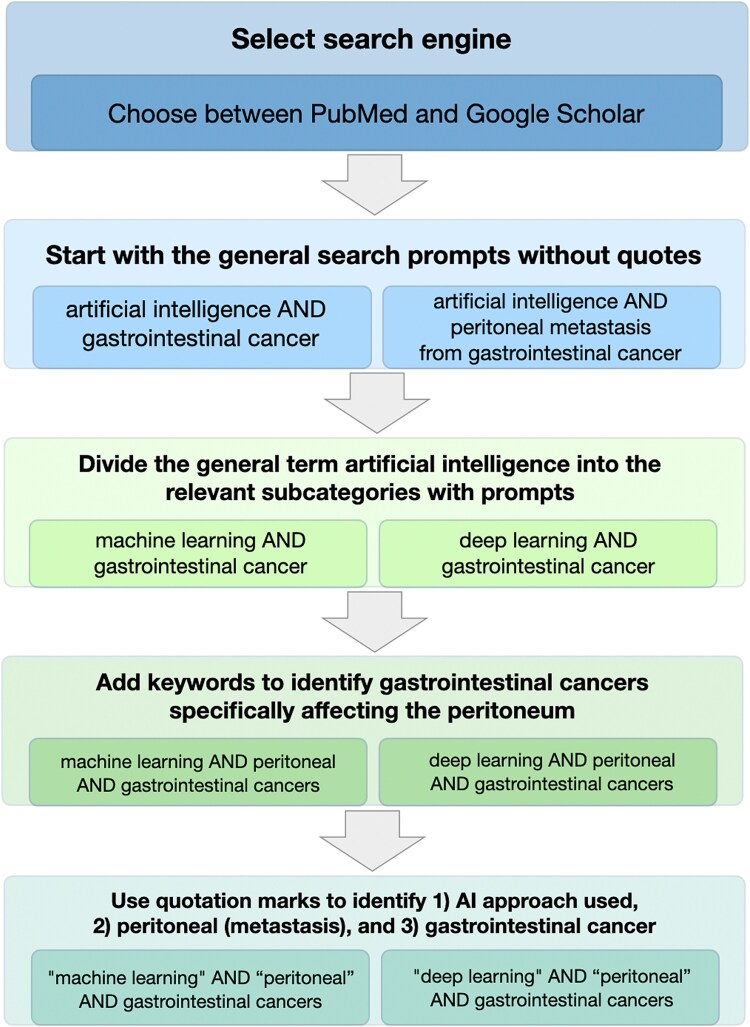
Literature search strategy including Boolean operators used. At the level of each arrow, the articles were examined for mentions of AI or statistical approaches used and results obtained. Abbreviation: AI, artificial intelligence.

The literature search included a comprehensive exploration of articles on PubMed for medical literature and on Google Scholar for academic literature. To maximize the searches yielding the relevant terms, Boolean logic was used to connect relevant terms. For quality control of this review, we restricted the articles consulted to only those on PubMed, ensuring that only published sources were consulted. The search queries used a combination of keywords selected to capture the intersection of ML, AI, and gastrointestinal cancers, with an emphasis on PM. The searches included queries such as *artificial intelligence* AND *gastrointestinal cancer* and *artificial intelligence* AND *peritoneal* AND *peritoneal metastasis from gastrointestinal cancer* (Fig. 2). Once the search results were displayed, individual articles were reviewed for content related to AI and/or statistics. We considered only articles with substantial detail about using AI in their experiments and with quantitative results, e.g. the area under the curve (AUC) of the receiver operating characteristic curve or the accuracy percentage. Articles lacking detailed methodology or quantitative performance metrics were excluded because their experimental outcomes could not be measured against other studies.

### Early detection of occult PM

Accurate prediction and early detection of occult PM are crucial for improving patient outcomes and guiding treatment strategies. Recent advancements in AI, particularly in DL techniques, have shown great promise in enhancing the accuracy and reliability of diagnosing PM. The following studies demonstrate how various AI models have been applied to predict occult PM, offering insights into their methodologies and performance.

Focusing on the predictions of occult PM among patients with advanced gastric cancer, Huang *et al.* [7] relied on deep convolution neural networks (DCNN) to support the preoperative diagnosis of occult PM (Table 1). This study elucidated image-acquisition procedures, data augmentation, and model development. The authors constructed their model input using manually annotated labels provided by a radiologist on the patients’ computed tomography (CT) images. The DCNN model was built with architecture featuring Xception with a Depthwise Convolution block and a Depthwise Separable Convolution within the block. This configuration yielded an AUC of 0.90, surpassing the performance of a clinical model *via* multivariate logistic regression (LR) (AUC of 0.67, Table 2). The model demonstrated robust generalizability across patients with varying ages and genders.

**Table 1:** AI and statistical models providing clinically relevant findings.

**Clinically relevant findings**	**Ref**
***Early detection of occult PM***	
Location of gastric tumor and Borrmann classification as independent predictors occult PM (age and gender neutral).	[7]
Intratumoral heterogeneity and Lauren type identified as important factors in PM from gastric cancer determination.	[8]
Association between preoperative tumor size, mild ascites, serum, and prediction of occult PM from gastric cancer.	[9]
Power of integrative approaches for predictions of occult PM in patients with advanced gastric cancer.	[11]
Distinctive features, such as area, perimeter, cytoplasm area fraction, cytoplasm area, and LD number were identified.	[12]
Comprehensive evaluation of multiple ML models.	[13]
***Detection and risk prediction of PM***	
Degree of nodularity, border transition, and degree of transparency as notable independent predictors of PM.	[15]
Importance of meta-analysis for PM prediction (gastric origin).	[16]
Albumins, platelet count, depth of infiltration, preoperative hemoglobin levels, weight for (gastric) PM detection.	[17]
Higher proportion of mucinous cell carcinoma in patients with PM. No significant impact of primary tumor site, sex, or age on detection of PM.	[18]
Age, gender, ascites, tumor size, differentiation status, and Borrmann classification for detection of PM (gastric origin).	[14]
Tumor size and T stage as independent factors predictive of (gastric) PM, recommendations for staging laparoscopy.	[13]
CT images of the primary gastric tumors identified to carry discriminatory information to predict the risk of PM.	[20]
Age, hematocrit, and hemoglobin parameters predictive of the presence of PM from colorectal cancer.	[23]
Primary tumor features and clinical variables important in the diagnosis of synchronous PM (colorectal).	[24]
***Forecasting PM recurrence***	
Disparity in pathomic signatures for predictions of predictions of (gastric) PM recurrence.	[25]
Rad-score and clinicopathological characteristic for predictions of (gastric) PM recurrence.	[26]
Linitis plastica, stump gastric cancer, tumor stage, lymph node involvement (pN2–3), and histological characteristics (Lauren classification and SRC histology) for (gastric) PM recurrence predictions.	[27]
Identification of the cohorts of patients to benefit from adjuvant systemic chemotherapy (gastric origin).	[30]
***Evaluating resectability from pre-operative data***	
The bowel involvement and pelvis involvement in predicting resectability (GI cancers).	[31]
The spatial distribution of PM among gastric origin patients, predominantly located in the upper and middle abdomen.	[32]
***Predicting complications after surgery***	
Insightful relationships between predictors and major complications (a risk score within 90 days post CRS-HIPEC).	[33]
Recommendation for surgical approach for patients with low-volume PM and a maximum of three resectable liver metastases originating from GI or gynecological primary tumors.	[35]
***Predicting survival after surgery***	
KPS score, resection type, and pretreatment platelet values were identified as the most important in predicting OS. In addition, age, sex, KPS score, tumor grade, tumor location, and TMN staging parameters as most important for the prediction of distant metastasis. KPS score, lymph node dissection type, tumor size, lymphatic invasion, pretreatment albumin and lymphocyte, tumor location, T or N stages, resection type, and concurrent chemotherapy as important variables to predict (gastric)PM recurrence.	[36]

**Table 2:** AI and statistical models used for detection and risk prediction of PM.

Approach^a^	Number of Samples	Data	Predicted Feature	Performance	Ref
DCNN, multivariable LR	544 patients Gastric cancer	CT Images	Occult PM diagnosis	AUC = 0.90	[7]
DCCN-LSC	1225 patients Gastric cancer	CT Images	Occult PM diagnosis	AUC = 0.96	[8]
Multivariate LR SVM, LR, MLP, RF	49 occult PM and 49 control patients Gastric cancer	CT-images Clinical data	Occult PM prediction	AUC = 0.85 (CT-clin) AUC = 0.62 (2D CT) AUC = 0.68 (3D CT)	[9]
BN, LR	810 patients Gastric cancer	CT-images Clinicopathological information	Occult PM prediction	AUC = 0.90–0.97 (clinicopathological) AUC = 0.91–0.98 (radiomics)	[11]
PCA, k-means, SVM, LDA, LR	27 p.m. positive 53 p.m. negative Gastric cancer	Imaged exfoliated cells (53,951)	Classification of PM	AUC = 0.80–0.85	[12]
DCNN Transfer learning	139 patient samples Gastric cancer	HE and PAP images	PM prediction	AUC = 0.89	[13]
CNN	87 lesions GI cancers	Laparoscopic images	Differentiate PM from lesions	AUC = 0.82	[15]
Radiomics Chi^2^ tests Fisher’s accurate test	6199 patients Gastric cancer	CT images Clinical data	Diagnostic performance of radiomics	AUC = 0.89	[16]
LR, DT, RF, GBDT, LightGBM	101 p.m. positive 979 p.m. negative Gastric cancer	Demographics Tumor characteristics	PM prediction	AUC = 0.94 (training) AUC = 0.74 (testing)	[17]
DCNN	131 patients Colorectal cancer Gastric cancer	Enhanced CT Images	PM diagnosis	AUC = 0.88	[18]
RF	239 Gastric cancer	IU and mixed images, Radiomics	PM prediction	Accuracy = 95% AUC = 0.98	[14]
Univariate LR Multivariate LR	498 patients Gastric cancer	CT images	PM detection	AUC = 0.93	[13]
GBM	159 Gastric cancer	CT Images	PM prediction	AUC = 0.69	[20]
ANN, SVM, RF	95 p.m. positive Colorectal cancer	Demographics Hematological indices	PM prediction	Accuracy = 76% AUC = 0.94	[23]
ResNet3D, SVM	74 p.m. positive 90 p.m. negative Colorectal cancer	CT images	PM detection	AUC = 0.92	[24]

^a^LR: logistic regression; SVM: support vector machine; RF: random forest; BN: Bayesian Network; DCNN: deep convolutional neural network; DCNN-LSC: densely connected convolutional network combined with long-short connections; PCA: principal component analysis; LDA: linear discriminant analysis; GBM: gradient boosting machine; ANN: artificial neural network; DT: decision tree; GBDT: gradient boosting decision tree; CNN: convolutional neural network; CT: computed tomography; IU: iodine uptake; PM: peritoneal metastasis; AUC: area under the curve.

Jiang *et al.* [8] also used DL to predict the occurrence of occult PM among gastric cancer patients. The training dataset included 1225 patients who underwent surgery. The validation dataset included 753 external patients who underwent surgery. In both cases, only patients with preoperative CT images were included for analysis. The authors developed a DCCN-LSC DL that included a (i) convolutional layer; (ii) two dense blocks, each followed by a transition layer; and (iii) a final dense block, followed by a pooling and a linear layer. Gradient-weighted class-activation mapping (Grad-CAM) was used to highlight image sections critical for the model-based diagnoses. The results yielded an AUC of 0.96 for the training dataset, and 0.95 and 0.92 for the two external validation cohorts. Interestingly, the class-activation maps generated by Grad-CAM highlighted variability in certain areas of the masked images. The variability in tumor regions suggested intratumoral heterogeneity as an important factor in determining PM. Moreover, apart from Lauren type, a consistent predictor of PM, integrating other clinical variables did not yield significant improvement over imaging data alone, underscoring the primary tumor’s importance in predicting PM.

Huang *et al.* [9] designed a comparative analysis of the performance between the clinical CT model and the 2D and 3D radiomics models in predicting occult PM in advanced gastric cancer. The study included a cohort of 1149 patients with advanced gastric cancer who underwent gastrectomy. First, the optimal features were determined using the algorithm for least absolute shrinkage and selection operator (LASSO) [10]. Then, the 2D- and 3D-radiomics models were constructed using ML algorithms like support vector machines (SVM), LR, multilayer perceptron, and random forest (RF). The clinical-CT model achieved an AUC of 0.85, outperforming both the 2D-radiomics model (0.62) and 3D-radiomics model (0.68). The authors attributed both radiomics models’ lower performances to tumor heterogeneity, suggesting that crucial information is not always captured by 2D texture analysis. The clinical-CT model showed promise by revealing a positive association between preoperative tumor size, mild ascites, serum, and PM.

Wang *et al.* [11] identified shortcomings in other ML studies. These limitations included modest AUC values and drawing conclusions from independent patient cohorts. To predict occult PM most effectively, Wang’s study enrolled 810 patients randomly assigned to three groups: 393 for training, 215 for internal validation, and 202 for external validation. Sixty-seven patients were diagnosed with occult PM. The patients’ data included clinicopathological information and CT images. With this dataset, the authors used the Bayesian network and LASSO LR to design a (i) radiomic signature model; (ii) a clinical model relying on clinicopathological data; and (iii) a model using both data sources. Across these models, the Bayesian network–based approach consistently yielded 0.90 to 0.97 AUC values for the clinical model and 0.91 to 0.96 for the radiomics model. These insights underscore the potential of integrative approaches in optimizing predictive models for occult PM for patients with advanced gastric cancer.

Chen *et al.* [12] developed a system reliant on ML approaches with AI-assisted–stimulated Raman molecular cytology; this system addressed the low sensitivity of current peritoneal lavage cytology. Like Su *et al.* [13], Chen *et al.* used various ML approaches across distinct tasks within their study; however, notable deviations were observed in the architecture of the model’s layers compared to the approaches of Su *et al.* Chen *et al.* extracted more than 50,000 exfoliated cells from ascites obtained from 80 gastric cancer patients (27 p.m.-positive and 53 p.m.-negative). Consequently, in place of the detection layer, the first layer of the model combined the principal component analysis (PCA) and k-means clustering (k-PCA) to generate clusters that could be used to generate marker cells, critical for the classification phase of the ML system. Supervised ML techniques, including SVM, linear discriminant analysis, and LR, were used for cell classification. Including the k-means clustering algorithm to generate groupings of cells according to the distances of their neighbors yielded substantial improvements; the same classification model’s performance transitioned from an AUC of 0.80 with SVM to an AUC of 0.85 with LR and k-means. Chen *et al.* highlighted that the efficacy of the integrated k-PCA approach successfully identified distinctive features, such as area, perimeter, cytoplasm area fraction, cytoplasm area, and LD number, despite significant disparities across clusters.

In the study by Su *et al.* [13], the authors carried out a combination of a DCNN with transfer learning. Their aim was predicting PM at the cellular level, enhancing cytopathology interpretation. While diagnosing PM using ascites data is feasible, laparoscopy and abdominal lavage fluids are typically required to verify the presence PM. Therefore, the authors focused on developing an approach that could yield better performance without executing unnecessary procedures. First, DetectionNet based on Region-Based CNN was developed to automatically detect all cells present in the input dataset, including the original hematoxylin–eosin and Papanicolaou-staining images of 139 patients. Subsequently, the ClassificationNet model extracted cell features and automatically classified each cell as malignant or benign. Among evaluated models, the DetectionNet ResNet18-based Faster R-CNN yielded the best mean average precision with an AUC of 0.83, while the ResNet50 showed greater performance in cell classification, with an AUC of 0.89.

### Detection and risk prediction of PM from imaging data

Insufficient sensitivity in detecting PM is a primary and well-recognized concern in CT-related research. Insufficient sensitivity lessens the value of CT analyses for informing clinical decision-making. To improve detection accuracy, explorations of more advanced imaging techniques or integrations of other diagnostic modalities, such as AI or radiomics, are needed. Indeed, the study conducted by Su [13] discussed in this section is the only study in this review that entirely relied on CT images and correctly diagnosed only 28% of positive CT results. Other modalities, such as radiomics, clinicopathological data, or a combination of both, were increasingly favored. Based on the performance of the models and as shown in the study by Chen *et al.* [14], further exploration of the combination of radiomics and clinicopathological data could determine whether this integration would yield better results. Applications of DL algorithms for predicting the presence of PM have demonstrated various efficiencies across studies. To distinguish PM from other types of peritoneal lesions, Schnelldorfer *et al.* [15] applied CNN to analyze the laparoscopic images of 87 peritoneal lesions (Table 2). Their analyses revealed that despite the availability of imaging data, a significant majority of peritoneal lesions remained misidentified. These incorrect diagnoses were due to a high rate of false positives, where lesions were erroneously identified as metastatic. In addition, a substantial number of actual metastatic lesions were overlooked. In response to these challenges, the authors proposed a DL model, which achieved an AUC of 0.82, exceeding the performance of the neural network (AUC = 0.47). Through their analysis, they identified characteristics such as the degree of nodularity, border transition, and degree of transparency as independent predictors of PM.

In the context of statistical approaches, acknowledging the meta-analysis conducted by Xue *et al.* [16] is important. This analyzed 10 studies focusing on the predictive capacity of radiomics for PM among gastric cancer patients. Including a cohort of 6199 patients across these studies, the comprehensive analysis revealed an overall AUC of 0.89. However, the considerable heterogeneity observed within these results suggested a lack of consistency in applying radiomics with other diagnostic modalities. Zhou *et al.* [17] comparatively analyzed the performances of five different ML algorithms to accurately predict PM among gastric cancer patients who underwent a preoperative CT scan and who were also CT-negative for PM. The algorithms included the following: LR, decision tree learning, RF, gradient boosting decision tree, and light gradient boosting machine (LightGBM). The patients considered had undergone preoperative CT scans, with CT results indicating a lack of PM. As opposed to previously discussed studies that relied on image data, Zhou *et al.* relied on text-based data comprising a patient’s (i) demographic details (age, sex, and body mass index); (ii) family history; (iii) tumor characteristics; and (iv) blood routine indices. The AUC for LightGBM was 0.94 (training set) and 0.75 (testing set). Results revealed that the five most influential factors contributing to the model’s predictions were the patient’s (i) albumin levels, (ii) platelet count, (iii) depth of infiltration, (iv) preoperative hemoglobin levels, and (v) weight, underscoring the potential of LightGBM as a robust tool for PM detection.

Zhang *et al.* [18] addressed the challenge of detecting PM from a different perspective, recognizing that current imaging modalities have a detection accuracy of only 29% for PM smaller than 0.5 cm. To tackle this limitation, the authors developed a DCNN. This study included 11,408 CT images from 131 patients, including 51 patients with PM and 80 without PM. The model developed in this study is noteworthy because it used metalearning techniques to predict PM. Metalearning enhanced the model’s generalizability by enabling it to simulate domain-shift scenarios. The model achieved an AUC of 0.73, surpassing the performance of existing methods for instances of PM with a size less than 0.5 cm. One additional finding highlighted was a higher proportion of mucinous cell carcinoma among patients with PM. Conversely, factors such as, primary tumor site, sex, and age, did not exert a significant impact on the model’s results, potentially because of the limitations imposed by the relatively small sample size.

Chen *et al.* [14] observed limitations in the current standard of using CT for detecting PM. They argued that CT can potentially misrepresent critical factors, such as tumor invasion depth and stage migration rate. To enhance PM-prediction accuracy, authors used a combination of dual-energy CT with an RF model. Images from patients with and without PM were preprocessed, resulting in the extraction of 1691 radiomics features from the peritoneal area and 1226 from the primary tumor. For dimensionality reduction, the Boruta [19] wrapper algorithm was applied to select features related to peritoneal status. The dataset, including different image types, was separated into three distinct groups: one containing iodine-uptake (IU) images; one with mixed images; and one with both. The RF algorithm tuned for IU images had the best performance in the training cohort, achieving an accuracy of 95% and an AUC of 0.98. Various clinical factors including age, gender, ascites, tumor size, invasion of tumor depth, differentiation status, and Bormann type, exposed a significant power in detecting PM.

Previous studies had limitations based either on insufficient sample sizes or on poor quality of CT images. In response to these limitations, the study by Su *et al.* [13] used 16- or 64-detector row scanners to measure the diagnostic performance of CT in detecting PM for patients with advanced gastric cancer. Out of the 1285 patients who underwent surgery for histopathologically confirmed gastric cancer, 498 met the inclusion criteria for the study. To determine the effectiveness of the CT images obtained, radiologists assigned one of three possible grades to indicate the presence of PM: grade 0 for PM absence; grade 1 for ambiguous presence; or grade 2 for PM presence. During the process, only 15 true-positive cases out of 53 actual cases (28.3%) of PM were identified. Subsequently, a comparative analysis between the 38 false-negative and 440 true-negative cases was conducted to identify risk factors of PM among patients classified as grade 0 or 1. Using univariate and multivariate LR, greater tumor size and T stage showed high confidence as independent factors predictive of PM. The model incorporating these variables together with four other significant variables yielded an AUC of 0.93. Because of the model’s poor performance in identifying grade 2 patients, typically associated with larger tumor size and T stage, the authors recommended staging laparoscopy, highlighting its importance in clinical decision-making.

Mirniaharika *et al.* [20] implemented a new computer-aided detection scheme that used ML approaches to predict the risk of PM for gastric cancer patients. The authors noted existing studies on radiomics-based ML models to differentiate gastric cancer patients with PM from those without PM. However, the data insufficiently represented the heterogenous characteristics of the tumors and limited prediction power of the models. With the computer-aided detection scheme, the authors adopted a more comprehensive approach, allowing 3D–tumor segmentation to extract 3D-image features. To simplify the data complexity, the authors explored five types of feature-reduction techniques, including (i) random projection algorithm, (ii) PCA, (iii) LASSO, (iv) minimum redundancy–maximum relevance [21], (v) and recursive feature elimination [22]. Combining the random production algorithm technique along with the gradient boosting machine model yielded superior performance metrics, reaching an accuracy of 71.2% and an AUC of 0.69. These outperformed models generated by previous studies by around 3%. The central observation was that the random production algorithm has potential as a tool for dimensionality reduction, and potential as a technique to generate optimal feature vectors for ML methods. These potentials underscore the significance of the random production algorithm in future research and of overcoming the need for laparoscopy.

### Detection and risk prediction of PM beyond imaging data

While the lack of interpretability in certain ML models remains a concern, many models, such as RF and regression models, provide valuable insights into feature significance, including imaging or nonimaging data. The comparative analyses of DL models discussed in this section offer important guidance for refining future models. A key finding is the strong influence of sample size on model performance. Models that integrate both ML and DL techniques have lower AUC values when trained on smaller datasets, underscoring the need for datasets with over 100 samples to achieve robust results. Generally, models trained on larger sample sizes, especially with image data, showed higher AUC values. However, given the limited number of studies using DL, further analysis is necessary to draw definitive conclusions. Additionally, integrating genomic data alongside clinical, demographic, and complete blood count information in AI models for PM detection and risk prediction remains underexplored and is central for advancing beyond imaging data alone.

Bejan *et al.* [23] developed ML algorithms, particularly methods involving artificial neural networks (ANN), SVM, and RF to predict the presence of PM among patients with colorectal cancer. The final datasets used by the authors included the comprehensive clinical information from a cohort of 95 patients with PM. This clinical information included patients’ demographics (sex and age) and hematological indices (hemoglobin levels, hematocrit, platelet count, white blood cell count, neutrophil count, and lymphocyte count). Random forest achieved the best accuracy of 76%, attributing its moderate performance primarily to three parameters: age, hematocrit level, and hemoglobin level.

A study conducted by Yuan *et al.* [24] similarly integrated ML and DL to detect synchronous PM in colorectal cancer. Using a systematic workflow and a comprehensive global analysis, the authors used preprocessing with contrast-enhanced CT scans. Samples covered 19,814 images in the training set and 7837 images in the testing set, which were obtained from 74 patients with PM and from 90 patients without PM. Subsequently, the data were analyzed using the ResNet-3D DL algorithm and passed into the SVM. The authors emphasized that integrating the models given the ResNet3D-SVM classifier resulted in an AUC of 0.92 and an accuracy of 94.11%, while the individual ResNet-3D model achieved an AUC of 0.76, and the SVM model achieved an AUC of 0.85. The integrated model elucidated the roles that primary tumor features and clinical variables play in diagnosing synchronous PM.

### Forecasting PM recurrence

Chen *et al.* [25] argued that existing methodologies were insufficiently focused on forecasting peritoneal recurrence among gastric cancer patients exhibiting serosal invasion (Table 3). To address this limitation, they developed a model for pathomics signatures using multiple features extracted from digital hematoxylin and eosin-stained images. Of the 341 patients included in the study, 206 were included in the training set, and 135 were included in the testing set. A total of 186 pathomics features were extracted using the STAR (Structured Transparent Accessible Reporting) methods, and pathomics signatures were constructed by the LASSO LR. Disparity in pathomics signatures was found between patients with and without peritoneal recurrence, and this disparity was the primary feature used by the model to generate the predictions. Across the training set, the AUCS were 0.85 (2 years), 0.85 (3 years), and 0.88 (5 years). Across the validation set, the AUCs were 0.87 (2 years), 0.89 (3 years), and 0.89 (5 years).

**Table 3:** AI and statistical models predicting PM recurrence, resectability, and complications post-surgery.

Approach^a^	Number of Samples	Data	Predicted Feature	Performance	Ref
LASSO LR	341 patients Gastric patients with serosal invasion	H&E images	Peritoneal recurrence	AUC = 0.87 at 2 years AUC = 0.89 at 3 years AUC = 0.89 at 5 years	[25]
SVM-RFE, LASSO, Cox regression, radiomics	433 patients Gastric cancer	CT images	Peritoneal recurrence	AUC = 0.68–0.85	[26]
Multivariate LR	645 patients Gastric cancer	Demographics Tumor characteristics	Peritoneal recurrence	AUC = 0.83	[27]
Multitask DL	Gastric cancer	CT images	Peritoneal recurrence Disease-free survival	AUC = 0.84–0.86 AUC = 0.71–0.75	[30]
DT, CTree, RF, SVM	310 GI cancers	Clinical data Demographics Etiological data	PM resectability	Accuracy = 97.82%	[31]
Various ML and DL models	168 p.m. patients Gastric cancer	CT images	Preoperative PM staging	AUC = 0.95 Accuracy = 91%	[32]
SHAP	2372 CRS patients	Clinicopathological data	Risk of major complications	AUC = 0.75	[33]
LR	>1000 CRS patients	Clinicopathological data	Severity of complications	AUC = 0.74	[34]
Univariate LR Kaplan–Meier Penalized Cox regression	37 patients Liver cancer	Clinicopathological information	Various predictions Morbidity related	Various	[35]
LR, MLP, XGBoost, SVM, RF, [24]	75 Gastric cancer	Clinical data	Overall survival Distant metastasis Peritoneal recurrence	AUC = 0.82 AUC = 0.87 AUC = 0.97	[36]

^a^LR: logistic regression; MLP: multilayer perceptron; XGBoost: extreme gradient boost; SVM: support vector machine; RF: random forest; GNB: Gaussian Naïve Bayes; DT: decision tree; CTree: conditional tree; LASSO: least absolute shrinkage and selection operator; SVM-RFE: SVM-Recursive Feature Elimination; SHAP: Shapley additive explanations; H&E: hematoxylin and eosin-stained images; CT: computed tomography; PM: peritoneal metastasis; AUC: area under the curve; CRS: cytoreductive surgery.

However, even for well-performing ML/DL models, the outcome related to predictive factors rather than to accurate predictions. Therefore, biostatistical methods should be integrated with and not alternative to ML or DL, yielding well-performing and transparent models. In line with that, Sun *et al.* [26] constructed a statistical model augmented by AI methods to predict peritoneal recurrence. This model used intramural- and peritumoral-radiomics features extracted from preoperative CT images from patients with gastric cancer after gastrectomy. The objective was to provide noninvasive biomarkers to augment clinical decision-making. The training set included 433 patients; the internal validation set included 417 patients; and the prospective validation set included 136 patients, all from the same hospital. Subsequently, the authors incorporated SVM–recursive feature elimination, LASSO, and the penalized Cox regression algorithm. The results for 1-, 3-, and 5-year PM-recurrence evaluations exhibited AUC values from 0.68 to 0.85. Similarly, the model showed robust performance in assessing disease-free survival. In addition to its predictive ability, the model identified patients eligible for adjuvant chemotherapy in stages II and III gastric cancer. By identifying low-risk patients who may not benefit from aggressive treatment, the model enabled clinicians to minimize the potential adverse effects of unnecessary interventions. On the contrary, high-risk patients can be selected for intensive treatment regiments and frequent monitoring, optimizing therapeutic outcomes.

Agnes *et al.* [27] introduced a prediction model to assess the risk of peritoneal recurrence following gastrectomy, and to establish a peritoneal recurrence index. After applying defined inclusion and exclusion criteria to an initial population of 1580 patients, 645 were selected for this study. The data collected included demographics, such as age and sex, and tumor characteristics, such as location and grade. To identify the variables most relevant for the prognostic assessment, a backward multivariable LR was conducted on the entire study cohort. Subsequently, two distinct models were developed. One model was based on the Lauren classification, which separates gastric adenocarcinomas into two primary subtypes: intestinal and diffuse [28]. The other model was based on the presence of signet ring cell features [29]. The resulting AUC value for the model based on the Lauren classification was 0.83, and the AUC value for the model based on signet ring cell features was 0.81. Analysis of the models highlighted that variables significantly associated with PM recurrence were (i) linitis plastica, (ii) stump gastric cancer, (iii) advanced tumor stage (pT3–4), (iv) extensive lymph node involvement (pN2–3), and (v) specific histological characteristics, such as the Lauren classification and signet ring cell histology. Agnes *et al.* claimed that the latter model holds promise in identifying a subgroup of patients who may benefit from proactive intraperitoneal prophylactic interventions, as well as those patients at high risk of PM.

Jiang *et al*, referenced earlier, introduced a different approach to assessing the risk of peritoneal recurrence. They created a model—based on CT images—that could simultaneously predict peritoneal recurrence and disease-free survival among patients [30]. The authors developed a DL network, integrating a supervised contrastive learning strategy with a dynamic neural network approach. The model yielded promising results, achieving an AUC of 0.84 to 0.86 for peritoneal recurrence and 0.71 to 0.75 for survival predictions. Beyond supporting the predictions of peritoneal recurrence and risk stratification among gastric cancer patients, the most significant benefit of this model was identifying patients who would most likely benefit from adjuvant systemic chemotherapy. The cohorts of these patients included stages II and III gastric cancer, with around 77% of patients with stage III, and 25% of patients with stage II.

### Evaluating resectability from preoperative data

Unlike prior studies, Maubert *et al.* [31] focused to predict the resectability of PM among patients potentially eligible for cytoreductive surgery and hyperthermic intraperitoneal chemotherapy (HIPEC) (Tables 1, 3). The authors used ML methodologies, such as conditional trees (CTree), RF, and SVM. The study cohort included 310 patients. This cohort consisted of 155 patients without resection and an equal number who underwent resection. Each patient’s dataset included epidemiological information and various surgical factors. Additionally, factors indicating organ damage, quantified using a Likert scale, were incorporated into the input parameters for the ML models. The RF model performed best, with an accuracy of 97.82%. Following closely was the SVM model, with an accuracy of 97.11%. The two variables with the greatest involvement in the RF model were bowel involvement and pelvis involvement in predicting resectability; whereas, ureter involvement was observed to be the least influential variable.

To stage PM preoperatively, Wang *et al.* [32] integrated ML and DL to analyze CT images from a cohort of 168 patients with PM. The data preprocessing steps included (i) normalization using Z-Score; (ii) feature selection with Spearman correlation coefficient; (iii) and regularization with LASSO. Radiomics and DL methodologies were then used to extract features of interest. Subsequently, a predictive model for PM index was developed, including various ML algorithms, such as LR, SVM, k-nearest neighbor, XGBoost, LightGBM, gradient boosting, RF, extra trees, and AdaBoost. The combination of DenseNet121 SVM returned the most effective model, achieving an AUC of 0.95 and an accuracy of 0.91 on the validation set. A key insight from this study was the spatial distribution of PM among gastric origin patients, predominantly located in the upper and middle abdomen. This insight suggested that further investigations of abdominal segmentation may offer valuable insights into PM dynamics across distinct groups of patients.

### Predicting complications after surgery

In the prognostic study by Deng *et al.* [33], an optimized ML model showed superior capability of predicting a risk of major complications after cytoreductive surgery with or without HIPEC compared to statistical methods (Tables 1 and 3). The final ML model achieved an AUC of 0.75. Shapley additive explanations dependence plots revealed insightful relationships between predictors and major complications. For example, elevated estimated blood loss proved detrimental only when the operative time passed 9 h. Predictions of severity of complications were also studied by Adam *et al.* [34]. Authors developed a risk score within 90 days after CRS-HIPEC among patients with PM. Several ML and traditional algorithms were considered. By defining comprehensive complication index scores ranging from 0 to 100 (median, 32.0), severe complications were observed for index scores greater than 61. The LR model achieved the highest performance (AUC, 0.74) and outperformed the NSQIP Surgical Risk Calculator. Factors positively associated with severe complications included, besides the PM index score, symptomatic status and undergoing pancreatectomy.

Horvath *et al.* [35] performed a retrospective analysis to assess the impact of various factors on the morbidity of PM patients undergoing liver resection and cytoreductive surgery plus HIPEC. The study considered 37 patients with available clinicopathological information. The authors performed Chi-squared tests and Fisher’s accurate tests to evaluate the relationship between distinct baseline properties and the incidence of postoperative complications. Univariate LR was used to determine which factors contributed to complications. Additionally, proportional hazard regression of Cox was used to evaluate the hazard ratio of these different risk factors. The central finding of the statistical analyses was that patients with low-volume PM and a maximum of three resectable liver metastases originating from gastrointestinal or gynecological primary tumors should be critically evaluated considering the surgical approach.

### Predicting survival after surgery

Akcay *et al.* [36] adopted the ML methods to evaluate overall survival (OS), distant metastasis, and peritoneal recurrence among patients diagnosed with gastric cancer. Their study cohort included 75 cases of gastric cancer patients who underwent radiation therapy ±CT. Before preprocessing, the initial dataset included a comprehensive collection of clinical parameters, including age, sex, Karnofsky Performance Scale (KPS), tumor characteristics, lymph node dissection details, TNM staging, histopathological features, and various hematological parameters. The Gaussian Naive Bayes algorithm resulted in the best performance for predicting OS, with an accuracy of 81.8% and an AUC of 0.82; the KPS score, resection type, and pretreatment platelet values were the values most responsible for its predictions. For predicting distant metastasis, the XGBoost algorithm emerged as the most effective, with an accuracy of 86% and an AUC of 0.86. Influential variables contributing to the model’s performance included age, sex, KPS score, tumor grade, tumor location, and TMN staging parameters. Lastly, the RF best predicted peritoneal recurrence with an accuracy of 97% and an AUC of 0.97; here, the important variables were KPS score, lymph node dissection type, tumor size, lymphatic invasion, pretreatment albumin and lymphocyte, tumor location, T stage, N stage, resection type, and concurrent chemotherapy.

## Discussion

PM presents a complex and challenging clinical problem. Traditional diagnostic and prognostic methods often underperform because of the nature of the disease, including its intratumoral and interpatient heterogeneity and its variability in response to treatments. As a solution, AI with statistical models offers a promising alternative to reveal patterns and make predictions. However, and as we show in this review, the performances of different models can vary significantly.

We identified several factors that prevent consistency. First, the performance of the models heavily depends on the preprocessing, quality, and amount of data as well as still-limited genomic sequencing. The size and quality of the training sets are crucial for developing robust ML models. Although larger datasets typically allow models to learn more effectively, for PM, obtaining large, diverse datasets can be challenging. Augmentation techniques and transfer learning can improve some of these limitations. Next, the variability in the quality, completeness, and consistency of these data sources can hinder model performance, i.e. missing data, outliers, and noise can significantly reduce model accuracy and reliability.

To mitigate these issues, oncologists should observe the following preprocessing techniques: proper imputation, normalization, and feature engineering. Feature selection is essential for ML models’ performance and outcomes. Importantly, different sets of features may be required for different models; e.g. conventional LR models will likely perform better with engineered features, while DL models will perform better with raw (image) data. Model complexity is another important factor to consider, and the trade-off between model complexity and interpretability, as well as model’s parameter set, must be carefully evaluated by a data scientist or an ML expert. Selection of performance metrics (commonly including accuracy, precision, recall, F1-score, or AUC) can be more relevant than other metrics depending on the specific objectives of the model and the characteristics of the dataset, particularly in cases of imbalanced data or varying costs of false positives and false negatives. Selecting metrics should be based on their relevance to the specific clinical question.

For example, *recall* is critical in clinical diagnoses to guarantee that as many positive cases as possible are identified, minimizing the risk of false negatives. In contrast, *precision* becomes more important when predicting treatment response, because accurately identifying true positives helps avoid unnecessary or incorrect treatments. The appropriate metric depends on the clinical context and the implications of potential prediction errors. Finally, *validation* of independent datasets allows testing and assessment of the generalizability and quality of ML models. Models that perform well on internal-validation datasets may not perform well on external datasets because of differences in patient populations, data collection methods, and clinical practices.

In summary of the models discussed in this review, no specific approach consistently outperformed any other, despite various applications and questions behind the model (e.g. prediction, recurrence, resectability, or survival). Even for a straightforward prediction of PM occurrence, which was the most frequent focus among the studies in this article, significant variations in the performance were observed. While RF emerged as a superior approach, its performance was not consistent. Therefore, including multiple models is justified, and researchers need to explore diverse ML methodologies. We also observed that the ML models can identify the most influential variables that impact model performance and, thus, impact decision-making.

## Conclusions and future directions

**Figure 3:**
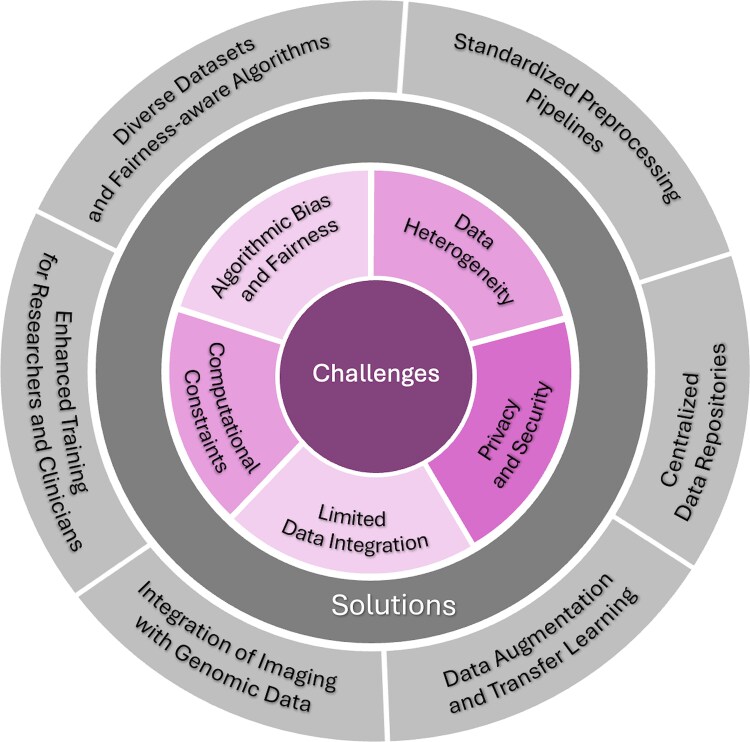
**Challenges and proposed solutions for integrating AI into personalized medicine and genomic studies of peritoneal metastases (PM) arising from gastrointestinal cancers.**  Inner Circle (Challenges): Core challenges specific to discussed AI approaches in PM include data heterogeneity, which impedes model generalizability across patient populations; privacy and security issues with sensitive medical data; algorithmic bias and fairness, leading to inequities in predictive outcomes; limited genomic data integration, restricting insights from multi-omics analyses; and computational constraints, which hinder scalability for large, complex datasets. Outer Circle (Solutions): Solutions proposed to address these barriers include standardized preprocessing pipelines for consistency and reproducibility, centralized data repositories to promote broader data sharing and diversity, data augmentation and transfer learning to overcome small dataset limitations, multi-institution collaborations to improve data heterogeneity and inclusiveness, and enhanced training for researchers and clinicians to bridge gaps between AI development and clinical application.

AI technologies hold promise for advancing current clinical practices in managing PM. By integrating multimodal data, such as imaging, genomics, and clinical information, ML models can significantly enhance diagnostic accuracy, enabling earlier and more precise detection of PM. AI can enable design of personalized treatments by analyzing extensive datasets to identify patterns and predict individual responses, optimizing therapeutic efficacy and minimizing adverse effects. Furthermore, and as discussed here, ML can predict PM progression and patient survival, offering valuable prognostic insights to clinical decision-making. For validating AI models, including the models we discuss in this review, future research should focus on large-scale, multicenter studies. These studies would allow for collecting diverse data, enhancing the generalizability and robustness of AI models across different patient populations and clinical settings. Collaboration across multiple institutions would also help in standardizing methods for data collection and allowing consistent results. Establishing partnerships with clinical practitioners is the key to ensure that AI models are not only technically rigorous but also practically applicable in clinical settings. Collaborations should focus on integrating AI tools into existing workflows, making them user-friendly for healthcare providers. Figure 3 emphasizes the interdependence of challenges and solutions, illustrating a pathway for advancing AI integration into PM research and personalized medicine for gastrointestinal cancers.

Integrating genomic data with statistics and AI models can significantly enhance predicting and managing PM by providing a comprehensive view of the disease at the molecular level. AI can further analyze a variety of biomarkers identified with statistical tools, including genetic mutations, gene expression profiles, epigenetic changes, and protein biomarkers. Specific genetic mutations can be identified to understand their role in PM prediction, development, or progression. Gene expression profiles can help identify the upregulated or downregulated genes in metastatic tissues, offering insights into the molecular mechanisms. Epigenetic changes, such as alterations in DNA methylation, can also be crucial because they influence gene expression without altering the DNA sequence. Additionally, changes in protein levels and post-translational modifications can indicate disease status and progression. By processing these diverse data types, AI models can uncover novel biomarkers and mechanisms, leading to improving diagnostic accuracy and to developing treatment strategies fitted to individual genetic and molecular profiles. Additionally, AI-driven analysis of genomic data can accelerate the identification of novel therapeutic targets and earlier detection of metastases, ultimately enhancing patient management and care.

**Summary key points**

- Artificial intelligence (AI) enables forecasting the development of peritoneal metastasis among patients with GI cancers.
- AI can provide insight into the nature of peritoneal metastasis.
- Because of the complex nature of peritoneal metastasis, choosing the right AI approach is key.

## Notes

**Adam Wojtulewski** is a Computer Science MSc student at the University of Florida. He joined the Combination Degree Program and specializes in artificial intelligence, including internship experience at Moffitt Cancer Center, Department of Machine Learning.

## Notes

**Aleksandra Sikora, MD** graduated from the Medical University of Warsaw in 2024. Her research and professional focuses are cardiology, oncology, and innovative methods for treating metastatic gastrointestinal cancers.

## Notes

**Sean Dineen, MD** is a surgical oncologist and assistant member of the Department of Gastrointestinal Oncology at Moffitt Cancer Center. His interest is in treating patients with metastatic disease, including carcinomatosis.

## Notes

**Mustafa Raoof, MD** is a surgical oncologist and an assistant professor in the Department of Surgery and the Department of Cancer Genetics and Epigenetics at City of Hope Cancer Center.

## Notes

**Aleksandra Karolak, PhD** is a computational chemist and data scientist in the Machine Learning Department at Moffitt Cancer Center. Her research focuses on analyses and predictions of cancer development, progression, and treatment.
